# Sex comparisons on the beneficial effects of an early intervention program in a patients’ cohort with first episode psychosis: what effectiveness in women?

**DOI:** 10.1007/s00737-025-01566-1

**Published:** 2025-02-19

**Authors:** Lorenzo Pelizza, Camilla Ricci, Emanuela Leuci, Emanuela Quattrone, Derna Palmisano, Simona Pupo, Giuseppina Paulillo, Clara Pellegrini, Pietro Pellegrini, Marco Menchetti

**Affiliations:** 1 Department of Biomedical and Neuromotor Sciences, Alma Mater Studiorum – Università di Bologna, Bologna, BO Italy; 2 Department of Mental Health and Pathological Addiction, Azienda USL Di Parma, Parma, PR Italy; 3 Pain Therapy Service, Department of Medicine and Surgery, Azienda Ospedaliero-Universitaria Di Parma, Parma, PR Italy

**Keywords:** First Episode Psychosis (FEP), Sex comparison, Women, Follow-up, Effectiveness

## Abstract

**Purpose:**

Males and females with First Episode Psychosis (FEP) usually tend to differ in psychopathology, clinical presentation and their longitudinal trajectory. This study aimed to examine the difference of effectiveness of specialized psychosocial and pharmacological treatments for FEP, focusing on various clinical and functioning outcomes across a 2-years follow-up period.

**Methods:**

The assessment included the CAARMS, the HoNOS, the PANSS and the GAF scale and was conducted at baseline and every 12 months.

**Results:**

490 FEP patients (age: 12–35 years) were recruited. Of them, 363 completed the follow-up (132 females and 231 males). At baseline, males showed a higher prevalence rate of schizophrenia diagnosis (56.1% VS 43.8%; *p* = .008), whereas females a higher prevalence rate of affective psychosis (36.2% VS 23.3%; *p* = .005). Male participants also showed a more consistent substance abuse (46.9% VS 24.3%; *p* = .0001), lower years of education (11.26 ± 2.94 VS 11.88 ± 2.68; *p* = .013), and more striking behavioral manifestations (4.06 ± 2.36 VS 3.39 ± 2.58; *p* = .003) compared to women. Our 2-year outcome parameter results showed a higher incidence of functional remission over time in females compared to males (49.2% VS 39.0%; *p* = .028), together with a decreasing trend in new hospitalization rates (17.8% VS 26.9%; *p* = .089). Independently from sex, our results also showed a statistically significant reduction in the prescription of psychotropic medications and through the increase of all psychosocial interventions, although more evident in males.

**Conclusion:**

These results suggested that specialized interventions for FEP are overall effective in both treated subgroups. Additionally, FEP women specifically showed higher rates of improvement in functional outcome variables over time when compared to males.

## Article Highlights

- Women with FEP showed less striking behavioral and clinical manifestations at entry compared to men.
- They also had a higher premorbid functioning and a higher prevalence rate of affective psychosis.
- The specialized interventions are overall effective in both treated sex subgroups, although more evident in males.
- However, women had a higher incidence of functional remission across the 2-year follow-up.

## Introduction

It is well known that *sex* differences may affect clinical course and prognosis of different mental disorders, particularly *psychosis*. Specifically, males and females with First Episode Psychosis (FEP) usually exhibit different in psychopathological features and clinical presentation from onset through their trajectory.

Males usually have a poorer *premorbid* functioning compared to females, especially in terms of work and school performance, independent living, social relationships, and socio-sexual development (Hafner 2003; Hui et al. 2016; Dama et al. 2019; Preston et al. 2002). At *presentation*, males often have an earlier age at onset, more severe psychopathology, higher severity levels of negative symptoms, and lower levels of current daily functioning, producing general poorer outcomes (Lindamer et al. 2003; Cotton et al. 2009; Thorup et al. 2014; Dama et al. 2019; Ayesa-Arriola et al. 2020; Carter et al. 2022; Preston et al. 2002). Males also show a higher prevalence of substance use disorder compared to women (Køster et al. 2008; Cotton et al. 2009; Arranz et al. 2015; Crosas et al. 2018; Ayesa-Arriola et al. 2020; Carter et al. 2022).

On the contrary, female sex is more commonly associated with better social functioning (Dama et al. 2019; Carter et al. 2022). They have an older age at entry, are more likely to be married and to be referred from general practitioner or polyclinics, and show higher treatment adherence (Pang et al. 2016; Ayesa-Arriola et al. 2020). However, women tend to have a longer duration of illness and to express more positive and depressive symptoms, including a more common history of suicide attempts at the entry (Køster et al. 2008; Austad et al. 2015). Evidence on Duration of Untreated Psychosis (DUP) was conflicting with some authors reporting no sex difference (Mattsson et al. 2007; Hui et al. 2016) while others observing longer DUP in males (Dama et al. 2019).

It was also reported that several cognitive domains are significantly correlated with the level of education and functioning in both sexes. Some studies found that males tend to have a better neurocognitive performance in the visual domain, working memory, and verbal performance than females (Hui et al. 2016; Danaher et al. 2018; Palacios-Garran et al. 2025). On the contrary, FEP females showed higher intelligence quotient and abilities in emotion recognition than males at presentation in EIP services (Ayesa-Arriola et al. 2020; Penney et al. 2023). Moreover, Ferrer-Quintero and co-workers (2022) notably reported a specific male socio-cognitive profile characterized by presenting jumping to conclusions and a specific female profile characterized by cognitive biases, suggesting that men and women could benefit from specific targeted cognitive treatment. This also highlights the need to consider sex when planning specialized EIP interventions.

Sex differences could also affect the *longitudinal course* of FEP, with females showing higher levels of social functioning (i.e., they more frequently continued living independently and had partners) and higher incidence to be in symptom remission and to be employed or in education (Dama et al. 2019; Ayesa-Arriola et al. 2020), whereas males more frequently tent to live alone, to suffer from substance abuse, and to be less compliant with therapy (with higher consequent rates of hospitalizations) (Cotton et al. 2009; Thorup et al. 2014). Previous investigations also reported that women had higher incidence of recovery over time and were more compliant with medication in comparison to men (Thorup et al. 2014). However, women’s positive attitude towards antipsychotic drugs has not been confirmed by more recent longitudinal studies (Zhou et al. 2016; Santos-Casado and García-Avello 2019), contributing to increasing inequalities in pharmacological treatment offer (Oehl et al. 2000). In this respect, the clinical trials of long-acting atypical antipsychotic medications showed that women are overall underrepresented.

As for clinical outcomes, positive and general symptoms often improve over time for both males and females. Females usually show greater improvement in negative symptoms than males, as well as in emotional and social withdrawal items and in poor impulse control behavior (Comacchio et al. 2019). Women also reported a better course of illness over a 1-year follow-up period, with higher improvement in positive symptoms, severity levels of psychopathology, disability and daily functioning (Pang et al. 2016; Dama et al. 2019). In this respect, Dama and co-workers (2019) found that women were more likely than men to exhibit good functioning after 1 but not after 2 years of treatment. However, these findings did not persist after controlling for other risk factors that could confound these associations (i.e., childhood premorbid functioning and age at onset of psychosis). The authors concluded that sex differences seen in outcomes among FEP patients treated in early intervention services for psychosis may be largely influenced by the disparity of other risk factors that exist between the 2 sexes. Likewise, in a 10-year observational research on 209 FEP patients previously treated in a specialized intervention service in Spain, Ayesa-Arriola and colleagues (2020) found that functioning and recovery differences did not show significant sex differences across the entire follow-up period. Specifically, according to the authors, the better outcomes seen among women during the first 3 years of treatment (i.e., while they were treated in the specialized service) were in the presence of more favorable premorbid and baseline characteristics. Indeed, after an average period of 10 years, outcomes for women approximated those of men. These findings help to pose the question of whether it is advisable to target sexes and lengthen early interventions.

Despite evidence on differences in prognosis between males and females with FEP, very few studies focused on longitudinal outcomes in relation to specialized “Early Intervention in Psychosis” (EIP) treatments. Different needs between men and women in terms of clinical profiles, illness trajectory and functional outcomes should be considered to develop more sex-specific therapeutic strategies (Chang et al. 2011; Carter et al. 2022). Finally, going deeper into the differences in symptomatology and cognitive deficits could facilitate personalized tailoring of interventions (Li et al. 2019; Dama et al. 2019).

For these reasons, our study aimed to examine baseline sex differences in terms of socio-demographic and psychopathological features and to investigate the difference in beneficial effects of specialized psychosocial and pharmacological treatments provided to a large cohort of both males and females with FEP, focusing on various clinical and functioning outcomes across a 2-years follow-up period.

## Materials and methods

### Setting and participants

All participants were enrolled within the “Parma Early Psychosis” (Pr-EP) program between January 2013 and December 2021. The PREP is a specialized EIP protocol diffusely implemented across all adolescent and adult mental healthcare services in the Parma Department of Mental Health (Northern Italy) (Pelizza et al. 2022a).

*Inclusion criteria* were: (a) specialist help-seeking, (b) enrollment in the Pr-EP program, (c) age 12–35 years, (d) presence of FEP within one of the following DSM-5 diagnoses: schizophrenia, bipolar disorder or major depressive disorder with psychotic features, delusional disorder, brief psychotic disorder, schizophreniform disorder, and psychotic disorder not otherwise specified (APA 2013), and (e) “Duration of Untreated Psychosis” (DUP) of < 2 years. A DUP length of < 2 years was selected in line with the usual time limit to provide effective interventions within the EIP paradigm (Woods et al. 2020). Although their clinical profiles could vary significantly, both affective and non-affective psychoses were included in this investigation. Indeed, the Pr-EP program is a real-world, non-academic setting, primarily engaged in the identification of optimal clinical care pathways for all FEP patients in standard community mental healthcare services (Pelizza et al. 2022b).

*Exclusion criteria* were: (a) history of previous overt psychotic episode (i.e., outside the current illness episode), (b) known intellectual disability (IQ < 70), and (c) neurological or other medical disease manifesting with psychiatric symptoms. Past exposure to antipsychotic medication (i.e., at any dosage and in previous illness episode before the Pr-EP enrollment) was considered as a “functional equivalent” of past psychotic episode (Pelizza et al. 2021).

All participants (including minors and their parents) provided their written informed consent for study participation. The research received local ethical approvals (AVEN Ethics Committee protocol n. 559/2020/OSS*/AUSLPR) and adhered to the 1964 Declaration of Helsinki (and its later amendments). The data are not publicly available due to privacy/ethical restrictions.

### Assessment

The psychopathological assessment included the “Comprehensive Assessment of At-Risk Mental States” (CAARMS) (Yung et al. 2005), the “Health of the Nation Outcome Scale” (HoNOS) (Wing et al. 1998), the “Positive And Negative Syndrome Scale” (PANSS) (Kay et al. 1987), and the “Global Assessment of Functioning” (GAF) scale (Aas 2011). This assessment was conducted both at baseline (T0) and every 12 months during the 2-year follow-up period by trained Pr-EP team members, using the approved Italian version of the scales.

The presence of FEP at entry was detected using the CAARMS psychometric criteria, approved Italian version. Regular CAARMS scoring workshops and supervision sessions were implemented to ensure good to excellent interrater reliability (Pelizza et al. 2019). Additionally, the baseline DSM-5 diagnosis was formulated by a minimum of two trained Pr-EP team members using the Structured Clinical Interview for DSM-5 mental disorders (SCID-5) (First et al. 2017).

The clinical and outcome assessment was based on the HoNOS, a widely used clinical instrument for evaluating mental health and social functioning in patients with severe mental illness, including young people with FEP (Belvederi Murri et al. 2023). As originally indicated (Wing et al. 1998), subscale scores were derived by grouping items into four main domains: (a) “Behavioral Problems”, (b) “Impairment”, (c) “Psychiatric Symptoms”, and (d) “Social Problems”. According to Kortrijk and co-workers (2012), we considered a score of ≤ 2 on the HoNOS items 9, 10 and 11 (all included in the HoNOS “Social Problems” domain) as index of *functional remission*.

The *PANSS* is a widely used clinical instrument for assessing psychopathology also in early psychosis (Leuci et al. 2022; Scazza et al. 2022). As proposed by Shafer and Dazzi (2019), we considered five principal psychopathological dimensions: “Disorganization”, “Negative Symptoms”, “Positive Symptoms”, “Resistance/Excitement-Activity”, and “Affect” (“Depression-Anxiety”). As index of current *symptomatic remission* across the follow-up period, we considered a score of ≤ 3 (corresponding to mild severity or less) on the 8 PANSS items indicated in the Remission in Schizophrenia Working Group’s criteria (Andreasen et al. 2005).

The *GAF* is a commonly used instrument for evaluating clinical status and socio-occupational functioning also in individuals with early psychosis. According to Yang and colleagues (2022), we considered a GAF score of > 60 at follow-ups as current index of *functional remission*. Specifically, both social and clinical GAF score of > 60 were considered to calculate the current index of functional remission.

Finally, a sociodemographic and clinical chart, including information on employment, DUP, new suicide attempt and self-harm behavior, current suicidal ideation, functional recovery, pharmacological therapy and service disengagement was completed both at entry and across the follow-up period, following the current definitions regarding suicide attempt (i.e., potentially injurious, self-inflicted behavior without a fatal outcome for which there was [implicit or explicit] evidence of intent to die, derived from direct information reported by the patient [or by a relative well informed about the facts], or documented in the clinical notes) (Schultze-Lutter et al. 2015; Silverman et al. 2007), service disengagement (i.e., complete lack of contact or untraceable for at least 3 months despite a need of treatment, counted from the date of the last face-to-face meeting with the clinical staff) (Robson and Greenwood 2022), current suicidal ideation (Pelizza et al. 2022c) and functional recovery (Silva and Restrepo 2019).

### Procedures

After CAARMS and SCID-5 interviews, FEP participants were divided into two subgroups based on sex. Between-group comparisons on sociodemographic, clinical and treatment measures at baseline were first investigated. The two subsamples were then examined for clinical outcomes with the HoNOS, the PANSS and the GAF interviews across the 2-year follow-up period. Moreover, 2-year incidence rates of service disengagement, new hospitalization, and new suicide attempt were calculated and compared between FEP males and females. Lastly, inter-group comparisons on treatment response were also investigated.

The Pr-EP program provided a 2-year treatment package including a psychopharmacological therapy and a multi-component psychosocial intervention (combining individual psychotherapy based on cognitive-behavioral approach, psychoeducational sessions for family members and a recovery-oriented case management) in accordance with the current EIP guidelines (NICE 2014; RER 2023).

### Statistical analysis

Collected data were analyzed using the Statistical Package for Social Science (SPSS) 15.0 for Windows (SPSS Inc 2010). All tests were two-tailed, with a significance level set at 0.05. In between-group comparisons, the Chi-square (Χ^2^) test for categorical variables and the Mann–Whitney U test for continuous measures were performed, using the Bonferroni’s corrected p values.

A mixed-design ANOVA was also carried out to investigate between-group comparisons on longitudinal psychopathological and outcome parameters, as well as for specialized Pr-EP treatment components. To allow the longitudinal examination of all these not time-to-event parameters, only FEP participants who concluded the whole follow-up period were included in our ANOVA tests.

As for time-to-event outcome parameters (e.g., service disengagement, new suicide attempt), after having previously checked that the proportionality-of-hazards assumption was met, univariate models were fitted for each outcome variable across the 2 years of follow-up, using Cox regression analysis (Zhang et al. 2018). In this case, as Cox regression analysis was able to take into account the different duration of follow-ups and participants who dropped out before the end of the study (Jager et al. 2008), all the FEP total sample was included in the statistical analysis. Finally, as for other not time-to-event dependent categorical parameters (e.g., current suicidal ideation, functional recovery, symptomatic remission), binary logistic regression analyses with sex as independent variable were performed exclusively in the FEP patients who ended the entire follow-up period (Harris 2021).

## Results

Over the course of this study, 490 FEP patients were recruited (305 males and 185 females). 53 participants dropped out the Pr-EP protocol during the first year of treatment and other 74 during the second year. At the end of our investigation, 363 FEP patients completed the follow-up (132 females [37.8%] and 231 males) (see Supplementary Materials [Figure S1] for details).

### Baseline data

Between-group comparisons on sociodemographic and clinical data at baseline showed that male participants were more likely to be migrants (males VS females: 58 [19.0%] VS 22 [11.9%]; *p* = 0.039) and had less years of education (males VS females: 11.26 ± 2.95 VS 11.88 ± 2.68; *p* = 0.013), more frequent referral to the Pr-EP program by family members (males VS females: 37 [12.1%] VS 12 [6.5%]; *p* = 0.043) and a higher prevalence rate of current substance abuse (males VS females: 143 [46.9%] VS 45 [24.3%]; *p* = 0.0001). Moreover, males showed a higher prevalence rate of schizophrenia as primary diagnosis at presentation (males VS females: 171 [56.1%] VS 81 [43.8%]; *p* = 0.008), whereas females showed a higher prevalence rate of affective psychosis (females VS males: 74 [23.3%] VS 67 [36.2%]; *p* = 0.005). Males also had a higher baseline PANSS “Resistance/Excitement-Activity” factor score (males VS females: 10.51 ± 5.07 VS 8.77 ± 4.07; *p* = 0.004), as well as higher baseline HoNOS “Behavioral Problems” domain score (males VS females: 4.06 ± 2.36 VS 3.39 ± 2.58; *p* = 0.003) (Table 1). As for specific psychopathological characteristics at entry, males showed higher scores in PANSS “Suspiciousness/Persecution” (males VS females: 4.34 ± 1.76 VS 3.91 ± 1.72; z = −2.082; *p* = 0.037), “Grandiosity” (males VS females: 2.21 ± 1.62 VS 1.58 ± 1.18; z = −3.683; *p* = 0.0001), “Hostility” (males VS females: 3.08 ± 1.81 VS 2.42 ± 1.35; z = −2.819; *p* = 0.005), “Poor impulse control” (males VS females: 2.96 ± 2.06 VS 2.43 ± 1.92; z = −2.509; *p* = 0.030), and “Lack of judgment/Insight” (males VS females: 3.48 ± 2.04 VS 2.90 ± 1.71; z = −2.176; *p* = 0.030) item subscores, while females a higher PANSS “Depression” item subscore (females VS males: 3.93 ± 1.66 VS 3.46 ± 1.67; z = −2.111; *p* = 0.035). As for the HoNOS features, the main inter-group differences regarded the higher “Aggression/Over-activity” (males VS females: 1.93 ± 1.30 VS 1.49 ± 1.22; z = −3.620; *p* = 0.0001) and “Substance abuse” (males VS females: 1.22 ± 1.29 VS 0.087 ± 1.20; z = −3.231; *p* = 0.001) item subscores in the male subgroup compared to the female one (see Supplementary Materials [Table S1] for details).

**Table 1:** Baseline sociodemographic and clinical comparisons in the two FEP subgroups (*n* = 490)

Variable	Males (*n* = 305)	Female (*n* = 185)	X2/z	p
Migrant Status Age (at entry) Education (in years) Civil status (single) Living status (living with parents) NEET	58 (19.0%) 25.59 ± 6.00 11.26 ± 2.94 202 (66.2%) 249 (81.6%) 163 (53.4%)	22 (11.9%) 25.10 ± 6.57 11.88 ± 2.68 111 (60.0%) 145 (78.4%) 96 (51.9%)	4.279 −1.252 −2.480 1.937 1.998.1. 111	**.039** .211 **.013** .164 .156 .739
Source of referral				
Primary care Family members Self-referral Emergency room School/Social services Other health services	91 (29.8%) 37 (12.1%) 21 (6.9%) 101 (33.1%) 18 (5.9%) 37 (12.1%)	68 (36.8%) 12 (6.5%) 15 (8.1%) 54 (29.2%) 7 (3.8%) 29 (15.7%)	2.516 4.077 .253 .821 1.067 1.241	.113 **.043** .615 .365 .302 .265
DUP (in months) Previous hospitalization Previous suicide attempt Substance misuse at entry Previous specialist contact	10.12 ± 10.41 137 (44.9%) 13 (4.3%) 143 (46.9%) 123 (40.3%)	9.40 ± 9.09 76 (41.1%) 14 (7.6%) 45 (24.3%) 78 (42.2%)	-.218 .690 2.416 24.780 7.160	.828 .406 .120 ** < .0001** .689
Baseline DSM-5 diagnosis				
Schizophrenia Affective psychosis Brief psychotic disorder Psychotic disorder NOS Schizophreniform disorder	171 (56.1%) 74 (23.3%) 42 (13.8%) 12 (3.9%) 6 (2.0%)	81 (43.8%) 67 (36.2%) 27 (14.6%) 9 (4.9%) 1 (0.5%)	6.954 8.029 .065 .243 1.664	**.008** **.005** .799 .622 .197
Baseline PANSS scores				
Positive symptoms Negative symptoms Disorganization Affect Resistance/Excitement-activity Total score Baseline GAF score	18.34 ± 6.10 24.28 ± 8.82 21.57 ± 8.14 15.93 ± 5.66 10.51 ± 5.07 94.15 ± 24.29 44.21 ± 9.96	16.97 ± 6.08 24.15 ± 8.59 19.99 ± 6.55 16.38 ± 5.18 8.77 ± 4.07 89.16 ± 21.81 45.15 ± 11.07	−1.879 -.317 −1.243 -.598 −2.896 −1.829 -.586	.060 .751 .214 .550 **.004** .067 .558
Baseline HoNOS scores				
Behavioral problems Impairment Psychiatric symptoms Social problems Total score	4.06 ± 2.36 3.23 ± 2.09 10.26 ± 3.26 7.80 ± 3.81 25.34 ± 8.35	3.39 ± 2.58 3.16 ± 2.17 10.04 ± 3.47 7.57 ± 4.05 24.15 ± 9.11	−2.978 -.462 -.713 -.916 −1.579	**.003** .644 .476 .360 .114
Baseline AP prescription Baseline LAI-AP prescription Equivalent dose of risperidone (mg/day) Baseline AD prescription Baseline MS prescription Baseline BDZ prescription Baseline individual psychotherapy proposal Baseline family psychoeducation proposal Baseline case management proposal	265 (86.9%) 34 (11.1%) 3.62 ± 2.55 45 (14.8%) 32 (10.5%) 110 (36.1%) 230 (75.4%) 189 (62.0%) 231 (75.7%)	153 (82.7%) 12 (6.5%) 3.26 ± 2.54 45 (24.3%) 34 (18.4%) 58 (31.4%) 143 (77.3%) 105 (56.8%) 137 (74.1%)	1.607 2.491 −2.138 7.034 6.145 1.136 .226 1.303 .175	.205 .086 **.033** **.008** **.013** .287 .635 .254 .676

FEP = First Episode Psychosis; NEET = Not [engaged] in Education, Employment or Training; DUP = Duration of Untreated Psychosis; DSM-5 = Diagnostic and Statistical Manual of mental disorders—5th Edition; PANSS = Positive And Negative Syndrome Scale; HoNOS = Health of the Nation Outcome Scale; AP = Antipsychotic medication; LAI-AP = Long-Acting Injection Antipsychotic medication; AD = Antidepressant medication; MS = Mood Stabilizer; BDZ = Benzodiazepine;. Frequencies (and percentages), mean ± standard deviation, Chi-square (X2) and Mann–Whitney U (z) test values are reported. Bonferroni’s corrected p values are reported. Statistically significant p values are in bold

Finally, female participants had higher baseline prescription rates of antidepressant and mood stabilizer medications, whereas males a higher baseline equivalent dose of antipsychotic drug (Table 1).

### Longitudinal data

**Fig. 1:**
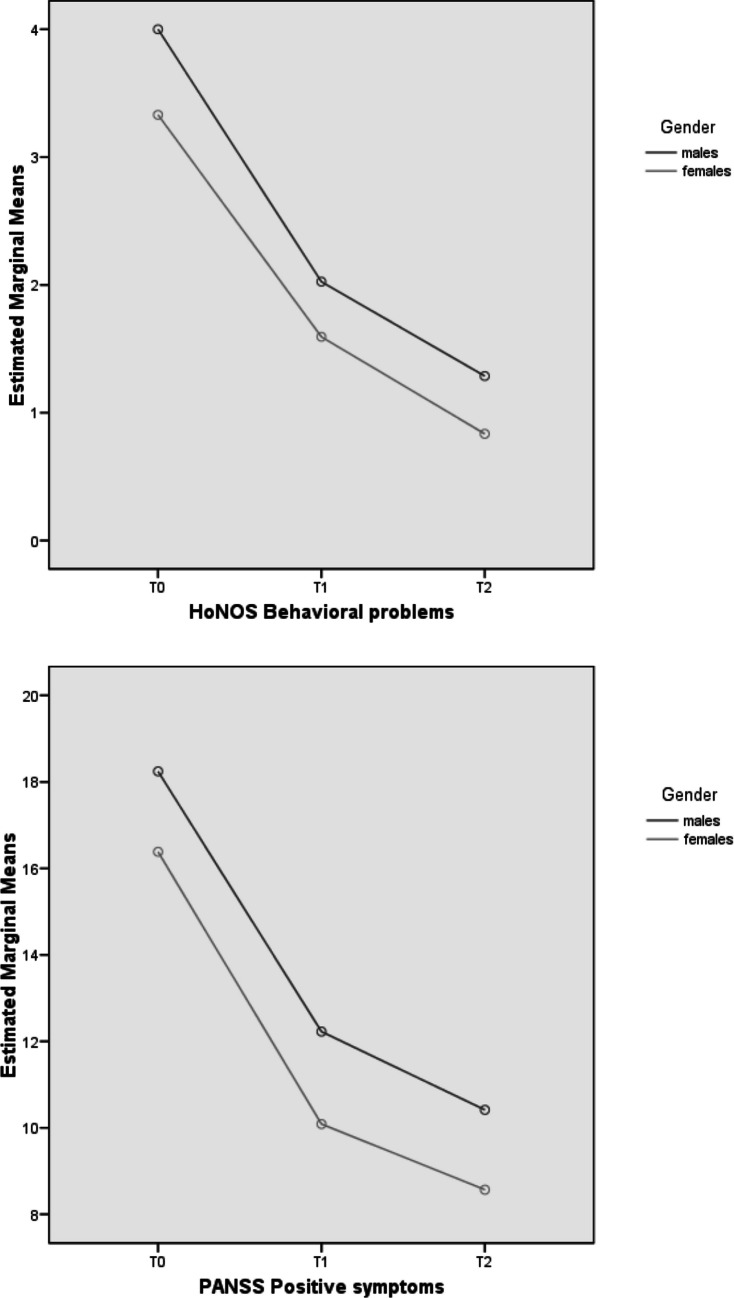

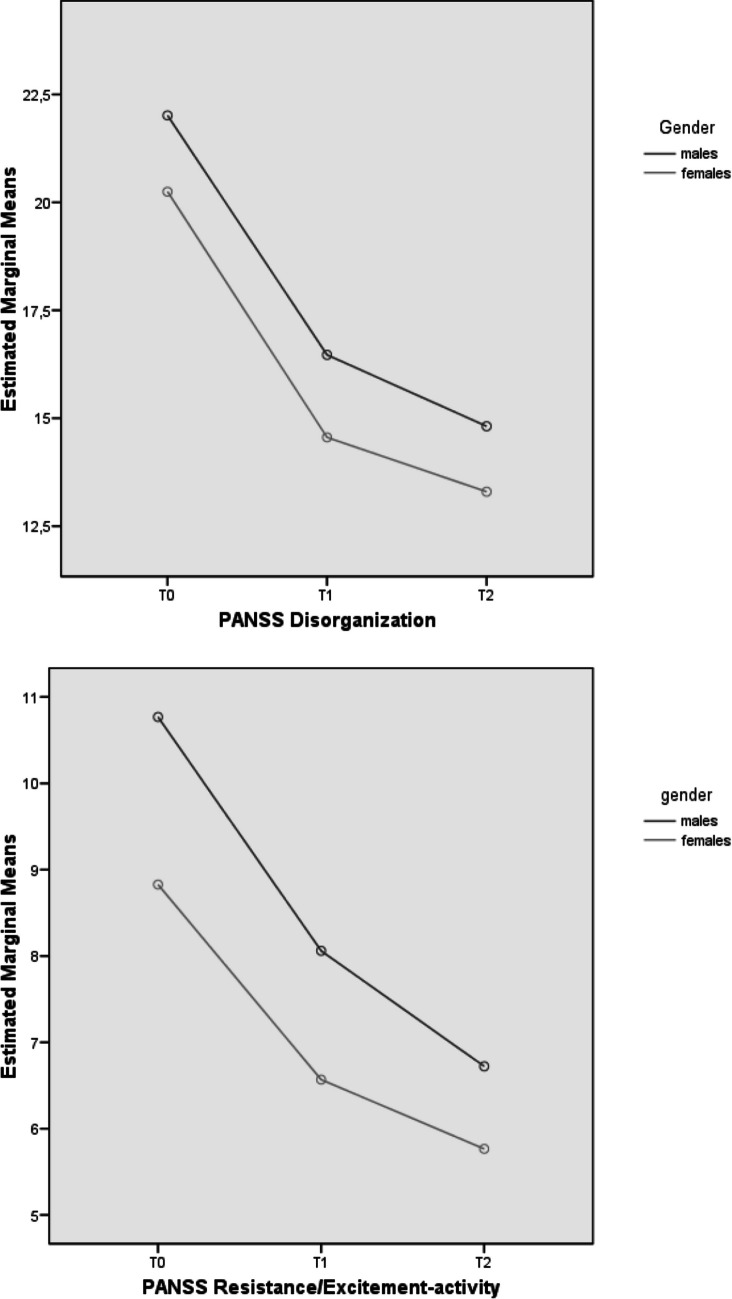

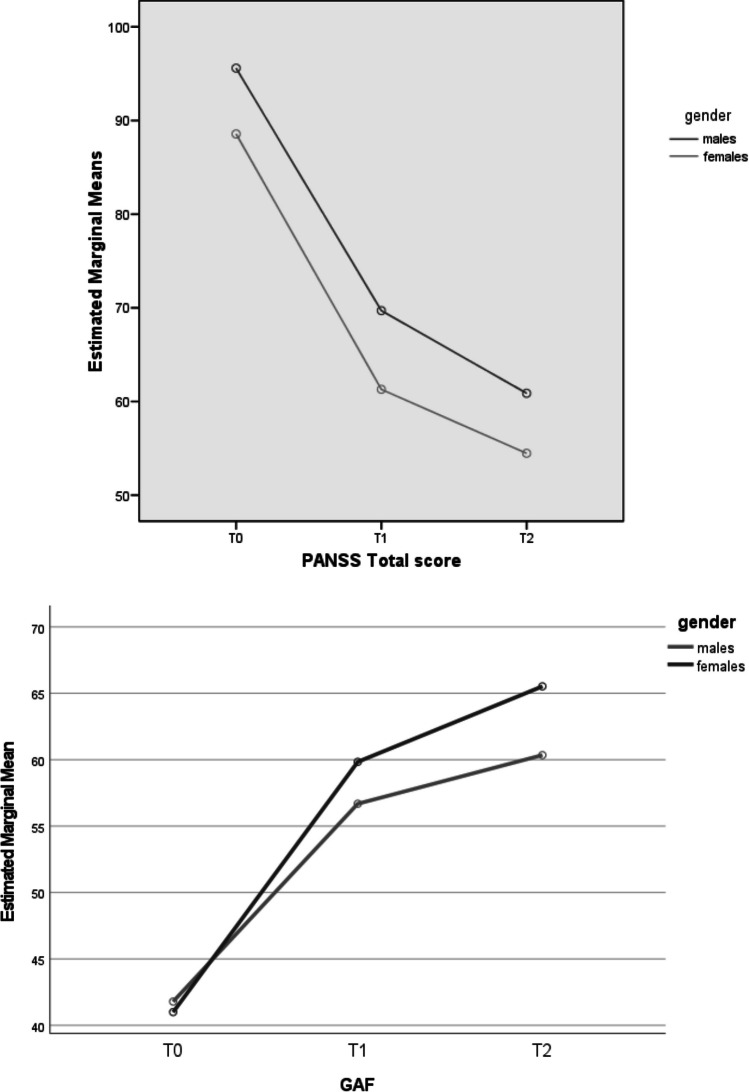
Mixed design ANOVA results: profile plots of statistically significant HoNOS scores across the 2-year follow-up period in the FEP total sample (*n* = 490). Note. ANOVA = Analysis of Variance; FEP = First Episode Psychosis; HoNOS = Health of the Nation Outcome Scale; PANSS = Positive And Negative Syndrome Scale; T0 = baseline assessment time; T1 = 1-year assessment time; T2 = 2-year assessment time

Our mixed-design ANOVA results showed a longitudinal improvement in all PANSS and GAF scores for both subgroups (Table 2). However, there was evidence of significant “group effects” for HoNOS “Behavioral Problems” domain scores and PANSS “Positive symptoms”, “Disorganization”, “Resistance/Excitement-activity” factor scores, as well as for GAF and PANSS total scores (see Fig. 1 for details on profile plots). Specifically, compared to males, females showed statistically relevant lower PANSS scores and higher GAF scores over time.

**Table 2:** Mixed-design ANOVA results: psychopathological and outcome parameters across the 2-year follow-up period in the two FEP subgroups

Variable	Time effect				Group effect				Interaction effect (time x group)			
	df	F	p	η2	df	F	p	η2	df	F	p	η2
HoNOS scores												
Behavioral problems Impairment Psychiatric symptoms Social problems Total score	1.6 1.5 1.7 1.6 1.6	258.423 347.922 427.366 225.784 538.835	**.0001** **.0001** **.0001** **.0001** **.0001**	.413 .340 .537 .380 .594	1 1 1 1 1	8.481 2.718 .387 1.713 3.415	**.004** .100 .534 .191 .060	.023 .007 .001 .005 .009	1.6 1.5 1.7 1.6 1.6	.623 1.341 .085 .396 .190	.507 .259 .890 .630 .775	.002 .004 .001 .001 .001
PANSS scores												
Positive symptoms	1.6	493.559	**.0001**	.498	1	9.015	**.003**	.038	1.6	.091	.872	.001
Negative symptoms	1.7	209.507	**.0001**	.425	1	2.618	.107	.011	1.7	.381	.648	.002
Disorganization	1.6	191.280	**.0001**	.455	1	4.510	**.035**	.019	1.6	.136	.824	.001
Affect	1.6	205.466	**.0001**	.471	1	.224	.636	.001	1.6	1.504	.225	.006
Resistance/Excitement-activity	1.4	99.688	**.0001**	.302	1	8.884	**.003**	.037	1.4	1.822	.174	.008
Total score	1.6	317.227	**.0001**	.582	1	6.476	**.012**	.028	1.6	.255	.732	.001
GAF score	1.6	182.977	**.0001**	.618	1	1.841	.178	.016	1.6	3.282	**.049**	.028
Variable (group effect)	EMM (SE)											
	Males (*n* = 231)	Females (*n* = 132)										
HoNOS score												
Behavioral problems	2.437 (.107)	1.920 (.142)										
PANSS scores												
Positive symptoms	13.627 (.383)	11.679 (.524)										
Disorganization	17.764 (.483)	16.033 (.657)										
Resistance/Excitement-activity	8.517 (.290)	7.053 (.396)										
Total score	75.394 (1.698)	68.111 (2.303)										
GAF score	52.944 (1.064)	55.456 (1.515)										

ANOVA = analysis of variance; FEP = First Episode Psychosis; HoNOS = Health of the Nation Outcome Scale; PANSS = Positive And Negative Syndrome Scale; GAF = Global Assessment of Functioning; df = degrees of freedom; F = F statistic value; *p* = statistical significance; η2 = partial eta squared; EMM = Estimated Marginal Mean; SE = Standard Error. As all Mauchly’s tests of sphericity are statistically significant (p < 0.05), Greenhouse–Geisser corrected degrees of freedom to assess the significance of the corresponding F value are used. Statistically significant p values are in bold. Only FEP participants who concluded the 2-year follow-up period are included in the analysis

**Fig. 2:**
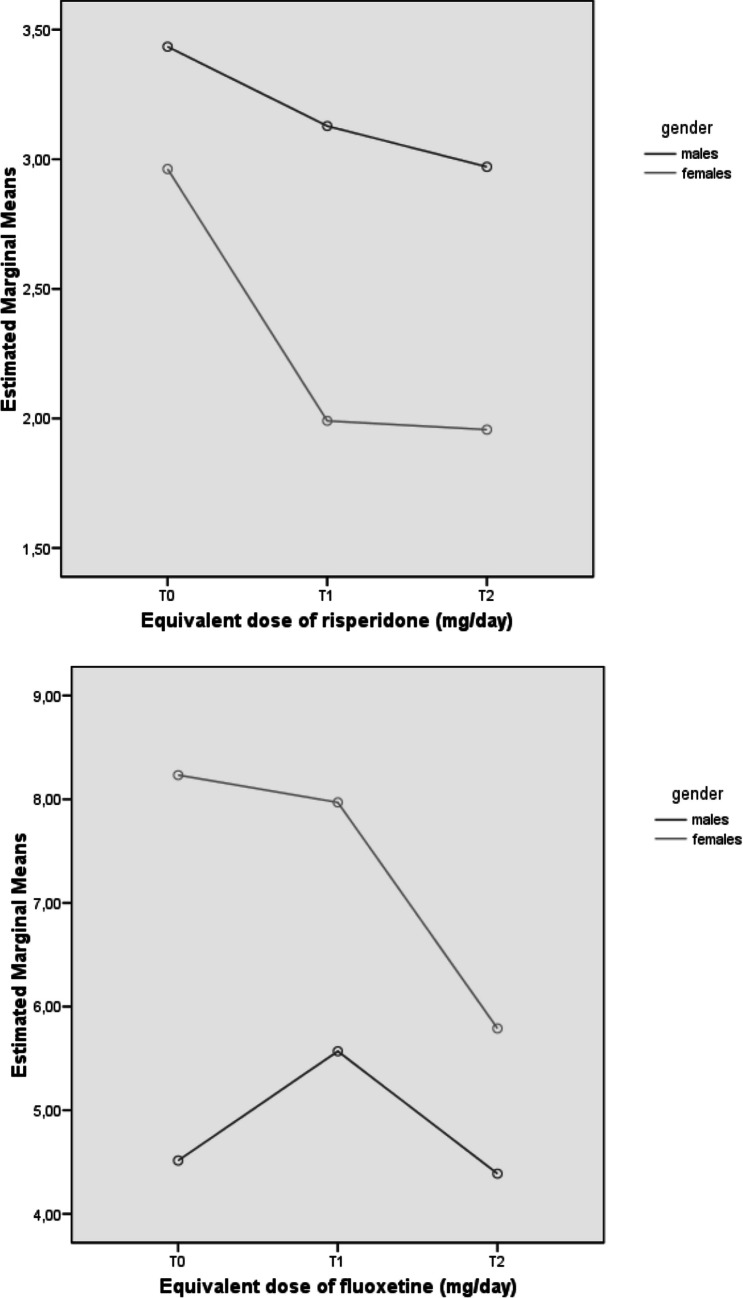
Mixed design ANOVA results: profile plots of statistically significant specialist treatment components of the Pr-EP program across the 2-year follow-up period in the FEP total sample (*n* = 490). Note. ANOVA = Analysis of Variance; Pr-EP = Parma Early Psychosis; FEP = First Episode Psychosis; T0 = baseline assessment time; T1 = 1-year assessment time; T2 = 2-year assessment time

As for specialized treatments provided in the Pr-EP program, there were evidence of a “time effect” with a significant longitudinal reduction in antipsychotic, antidepressants, and mood stabilizer dosages as well as a relevant increase in all psychosocial interventions. The most significant result was the decrease in antipsychotic dosage, showing not only a “time effect”, but also a “group” and an “interaction effect” (Table 3 and Fig. 2). Specifically, the female subgroup overall showed lower antipsychotic doses across the entire follow-up period and a higher decrease in their dosage over time (especially in the first year of treatment). Additionally, women reported higher antidepressant doses across the whole follow-up period than men. No group differences were found in terms of intensity in Pr-EP psychosocial interventions.

**Table 3:** Mixed-design ANOVA results: specialized treatment components of the Pr-EP program across the 2-year follow-up period in the two FEP subgroups (*n* = 490)

Variable	Time effect				Group effect				Interaction effect (time x group)			
	df	F	p	η2	df	F	p	η2	df	F	p	η2
Equivalent dose of risperidone (mg/day)	1.7	16.848	**.0001**	.044	1	12.358	**.0001**	.033	1.7	3.307	**.044**	.009
Equivalent dose of fluoxetine (mg/day)	1.6	3.982	**.026**	.011	1	5.807	**.025**	.014	1.6	1.741	.182	.005
Equivalent dose of lorazepam (mg/day)	1.8	6.267	**.003**	.017	1	.875	.350	.002	1.8	2.509	.089	.007
Equivalent dose of lithium (mg/day)	1.5	.931	.371	.003	1	.737	.391	.002	1.5	2.416	.105	.007
Individual psychotherapy sessions	1.3	428.937	**.0001**	.468	1	.964	.327	.002	1.3	.746	.423	.002
Family psychoeducation sessions	1.2	268.251	**.0001**	.422	1	.773	.380	.002	1.2	.668	.445	.002
Case management sessions	1.2	348.809	**.0001**	.487	1	.101	.751	.001	1.2	.349	.590	.001
Variable (group effect)	EMM (SE)											
	Males (*n* = 231)	Females (*n* = 132)										
Equivalent dose of risperidone (mg/day)	2.303 (.199)	3.178 (.149)										
Equivalent dose of fluoxetine (mg/day)	4.824 (.666)	7.331 (.889)										

ANOVA = analysis of variance; Pr-EP = Parma Early Psychosis; FEP = First Episode Psychosis; df = degrees of freedom; F = F statistic value; *p* = statistical significance; η2 = partial eta squared. As all Mauchly’s tests of sphericity are statistically significant (p < 0.05), Greenhouse–Geisser corrected degrees of freedom to assess the significance of the corresponding F value are used. Statistically significant p values are in bold. Only FEP participants who concluded the 2-year follow-up period are included in the analysis

Our univariate Cox proportional-hazard model results for 2-year time-to-event outcome parameters (including attrition rates) showed a higher 2-year new hospitalization incidence rates in males than in the female subgroup. No other statistically significant differences between male and female subgroups were observed (Table 4).

**Table 4:** Univariate Cox proportional-hazard models for 2-year time-to-event outcome parameters in the two FEP subgroups (*n* = 490)

Variable	Males (*n* = 305)	Females (*n* = 185)	Statistic test			
			HR	95% CI		*p*
				Lower Higher		
2-year service disengagement	74 (24.3%)	53 (28.6%)	.808	.568	1.149	.235
2-year new hospitalization	90 (29.5%)	33 (17.8%)	1.419	.948	2.126	**.029**
2-year new suicide attempt	9 (3.0%)	11 (5.9%)	.467	.193	1.127	.090
2-year new self-harm behavior	31 (10.2%)	15 (8.1%)	1.180	.637	2.186	.599

FEP = First Episode Psychosis; HR = Hazard Ratio; 95% CI = 95% confidence intervals for HR; *p* = statistical significance. Significant statistical p values are in bold. All FEP participants are included in the analysis

Service disengagement = complete lack of contact or untraceable for at least 3 months despite a need of treatment, counted from the date of the last face-to-face meeting with the clinical staff; Suicide attempt = potentially injurious, self-inflicted behavior without a fatal outcome for which there was (implicit or explicit) evidence of intent to die, derived from direct information reported by the patient (or by a relative well informed about the facts) or documented in the clinical notes; Self-harm behavior = acts of deliberate self-harm or intoxication with alcohol or drugs, but where there was no clear intention to die

Our binary logistic regression analysis results for 2-year not-time-to-event outcome variables showed that female participants had a significant higher 2-year incidence rate of HoNOS functional remission compared to males, whereas males had a higher 2-year incidence rate of new hospitalization compared to females (Table 5).

**Table 5:** Binary logistic regression analysis results for 2-year not time-to-event outcome variables in the two FEP subgroups

Dependent variable	Males (*n* = 231)	Females (*n* = 132)	Statistic test				
			B (SE)	HR	95% CI		*p*
					Lower Higher		
2-year current suicidal ideation	43 (18.6%)	22 (16.7%)	-.133 (.246)	.876	.541	1.417	.589
2-year functional recovery	157(67.9%)	98 (74.2%)	.314 (.209)	1.369	.909	2.062	.132
2-year GAF functional remission	138 (59.7%)	90 (68.2%)	.367 (.196)	1.443	.982	2.210	.062
2-year HoNOS functional remission	90 (38.9%)	65 (49.2%)	.414 (.188)	1.513	1.046	2.188	**.028**
2-year PANSS symptomatic remission	139 (60.1%)	86 (65.1%)	.208 (.193)	1.231	.843	1.798	.283
2-year persistent negative symptoms	20 (8.6%)	9 (6.8%)	-.209 (.353)	.811	.406	1.621	.553
2-year new hospitalization	67 (29.0%)	23 (17.4%)	-.527 (.231)	.590	.375	.929	**.023**
2-year new suicide attempt	7 (3.0%)	8 (6.1%)	.732 (.460)	2.079	.845	5.117	.111
2-year new self-harm behavior	23 (9.9%)	11 (8.3%)	-.249 (.329)	.780	.409	1.487	.450

FEP = First Episode Psychosis; GAF = Global Assessment of Functioning; HoNOS = Health of the Nation Outcome Scale; PANSS = Positive And Negative Syndrome Scale; B = regression coefficient, SE = Standard Error; HR = Hazard Ratio; 95% CI = 95% confidence intervals for HR; *p* = statistical significance. Significant statistical p values are in bold. Cumulative incidence rates are reported. Only FEP participants who concluded the 2-year follow-up period are included in the analysis

Current suicidal ideation = BPRS item 4 score ≥ 2; Functional recovery = return to work/school; GAF functional remission = GAF score ≥ 60; HoNOS functional remission = HoNOS item 9, 10 and 11 subscores < 2; PANSS symptomatic remission = PANSS item P1, P2, P3, N1, N4, N6, G5, G9 subscores ≤ 3; Persistent negative symptoms = (a) presence of at least moderate (i.e., a score of 4 on the PANSS) for at least 3 negative symptoms or at least moderately severe (i.e., a score of 5 on the PANSS) for at least 2 negative symptoms + (b) persistence of negative symptoms for at least 6 months and for an extended period of time prior to the study beginning (e.g., at least 4 weeks) + (c) absence of relevant levels of positive symptoms, depression and extrapyramidal symptoms; Suicide attempt = potentially injurious, self-inflicted behavior without a fatal outcome for which there was (implicit or explicit) evidence of intent to die, derived from direct information reported by the patient (or by a relative well informed about the facts) or documented in the clinical notes; Self-harm behavior = acts of deliberate self-harm or intoxication with alcohol or drugs, but where there was no clear intention to die

## Discussion and conclusions

The aim of this investigation was to examine baseline sex differences in terms of socio-demographic and psychopathological features and to explore the difference in beneficial effects of specialized psychosocial and pharmacological treatments provided to a large cohort of both males and females with FEP, focusing on various clinical and functioning outcomes across a 2-years follow-up period. We had no specific a-priori hypotheses. Our investigation was an observational description of potential sex differences in terms of baseline clinical features and longitudinal outcomes in order to evaluate new confirming or conflicting data in comparison with little research evidence on this topic published in the current literature to date.

The higher prevalence of males (62%) than females (38%) at entry confirms the younger age of psychosis onset in males and the need to extend the age range of inclusion criteria to access EIP services beyond 35 years in order to improve specialized care for women (White 2011; Ferrara and Srihari 2021; Ayesa-Arriola et al. 2020; Ferrara et al. 2024; Naughton et al. 2024). In this respect, females with FEP have specific health needs that may be proactively addressed to refine the current expansion of EIP services worldwide, so that the historical *underrepresentation of women* in using these services can be overcome. Moreover, it should also be noticed that a portion of this male prevalence was due to a more frequent status of migrants. In this respect, it is more common to meet young males alone in search of a better life and anticipating the journey of their family members (Spagnoli et al. 2020). Implementing mediation services for migrants with FEP within EIP programs is therefore crucial to improve treatment outcomes, especially in males (Tarricone et al. 2012).

As for baseline DSM-5 diagnoses, our results are in line with previous findings, with males more frequently affected by schizophrenia and females by *affective psychosis* (Køster et al. 2008; Comacchio et al. 2019; Ayesa-Arriola et al. 2020). This also seems to justify the greater prescription rates of antidepressant and mood stabilizers at entry in women compared to men, who had higher baseline doses of antipsychotic medication than females. Moreover, this further justifies the older age of psychosis onset in females and the need to extend the age range of inclusion criteria to access EIP programs beyond 35 years in order to include later psychosis onset (especially in terms of affective psychosis) (Ramain et al. 2022).

Concordantly with previous results (; Køster et al. 2008; Cotton et al. 2009; Chang et al. 2011; Crosas et al. 2018; Ayesa-Arriola et al. 2020), our male participants also showed a more consistent *substance abuse* at presentation compared to FEP females, as well as lower *years of education*. Together with higher scores in males on HoNOS “Behavioral Problems” domain and PANSS “Resistance/Excitement-Activity” dimension, our findings further support the presence of more striking *behavioral manifestations* at entry (including higher levels of suspiciousness/persecution and grandiosity) and a lower premorbid functioning (especially in terms of worse school performance) in men with FEP compared to women (Køster et al. 2008; Thorup et al. 2014; Arranz et al. 2015; Irving et al. 2021; Dama et al. 2019; Ayesa-Arriola et al. 2020). Coupled with higher levels of lack of judgment/*insight* at presentation in our male participants, these findings could also justify a more frequent referral by family members in men than female patients, as well as a lower *care collaboration* in males with FEP right from the Pr-EP enrollment.

In this investigation, women with FEP differently showed a higher baseline PANSS “*Depression*” item subscore, in line with their greater prevalence of affective psychosis at entry, suggesting clinical manifestations more oriented on the affective side (Chang et al. 2011; Ochoa et al. 2012; Comacchio et al. 2019; Irving et al. 2021; Ayesa-Arriola et al. 2020).

As for longitudinal results, the evidence of time effects within our total FEP population supports the effectiveness of specialized EIP interventions provided in the Pr-EP program overtime in both males and females. The evidence of a group effect in HoNOS “Behavioral problems” and PANSS “Positive symptoms”, “Disorganization”, “Resistance/Excitement-Activity” factor scores, as well as for PANSS total score, seems to support a more marked clinical severity across the follow-up period in the male subgroup compared to females, despite a general clinical improvement in both groups. Additionally, the time x group effect in GAF total score could further supports a higher longitudinal improvement in functioning in FEP women, in line with previous results reported in international perspective research (Mattsson et al. 2007; Køster et al. 2008; Cotton et al. 2009; Chang et al. 2011; Ochoa et al. 2012; Thorup et al. 2014; Dama et al. 2019; Ayesa-Arriola et al. 2020; Preston et al. 2002).

Further findings supporting the Pr-EP program’s beneficial effects, independently from sex, were given by the evidence of a statistically significant longitudinal reduction in the prescription of psychotropic medications, as well as through the increase of all psychosocial interventions over time. However, the group effect of the antidepressant treatment seems further confirm the more frequent affective nature of FEP in women. Moreover, the interaction effect in the antipsychotic treatment may reflect more severe longitudinal levels of psychopathology in men and/or a greater caution when prescribing antipsychotics in women. On the contrary, between their 3- and 10-year follow-up sessions, Ayesa-Arriola and co-workers (2020) observed an increase in dosage of antipsychotics in the female subgroup. Finally, in this research, no sex difference was found in terms of intensity of psychosocial interventions provided within the Pr-EP program.

Our 2-year outcome parameter results showed a higher incidence of functional remission over time in females compared to males. These results appear to be in line with the current literature, showing the evidence of a better general recovery in FEP women compared to men, especially in the first year of treatment (Cotton et al. 2009; Hui et al. 2016; Pang et al. 2016). This finding may be explained also by the more common evidence of behavioral disorders, lack of insight, and comorbidity with substance abuse in men (Cotton et al. 2009; Pang et al. 2016; Carter et al. 2022), which could also influence higher incident rates of new hospital admissions (Crosas et al. 2018). Nevertheless, the comorbidity with substance abuse seems not to be the main reason of the worse clinical condition of male patients, with clear intrinsic sex differences which could have a substantial influence on the clinical presentation of FEP (Irving et al. 2021).

### Limitations

The present study has several strengths, although some limitation should be considered. Firstly, the sample size was quite consistent; however, there was a part of the total FEP population who dropped out the study before the end of the follow-up (25.9%).

Secondly, this study considered only the socio-demographic and clinical differences of the two groups, whereas any cognitive difference at baseline was not explored, as was reported in other studies (Hui et al. 2016; Li et al. 2019; Vila-Badia et al. 2021).

Furthermore, the current research was conducted across 2 years of follow-up, whereas future studies with a longer observational period should be needed to better define the differences in outcomes between men and women. Moreover, our examination on not time-to-event parameters did not take into account for attrition rate and missing values. This may limit the generalizability of our findings.

Additionally, without a control group, this investigation was not able to explore the real effectiveness of FEP treatments considering sex. Rather, we provided a descriptive account of program outcomes. Therefore, future randomized control trials with control groups are needed.

Finally, we did not examine whether the reported differences in outcome parameters might lead to the need to develop a differentiated type of psychosocial or pharmacological intervention between men and women (Køster et al. 2008; Chang et al. 2011). This will be deeper explored in future investigations.

### Conclusions

The results of our study suggested that the Pr-EP program interventions overall have beneficial effects in both treated subgroups (including FEP females). Additionally, FEP women specifically showed higher rates of improvement in functional outcome variables over time when compared to males. This undoubtedly extends the clinical usefulness of specialized EIP interventions for females also into the context of Italian public mental healthcare services. Specifically, our results extend beneficial effects of treatments for women to the entire EIP period (i.e., 2 years) and pose the question of whether it is advisable to lengthen early interventions in females. In this respect, recent follow-up studies (up to 10 years of observation) did not show significant sex differences in terms of functioning and recovery over time, especially once early intervention was completed (Dama et al. 2019; Ayesa-Arriola et al. 2020). Moreover, further studies should be conducted to better define the implications that these findings may have on possible sex-specific treatments.

## Data Availability

The data that support the findings of this research are available on reasonable request from the corresponding author. The data are not publicly available due to privacy and/or ethical restrictions.
